# Qualitative exploration of facilitating factors and barriers to use of antenatal care services by pregnant women in urban and rural settings in Pakistan

**DOI:** 10.1186/s12884-016-0829-8

**Published:** 2016-03-01

**Authors:** Yasir Bin Nisar, Brekhna Aurangzeb, Michael J Dibley, Ashraful Alam

**Affiliations:** Sydney School of Public Health, The University of Sydney, Sydney, NSW 2006 Australia; Children’s Hospital, Pakistan Institute of Medical Sciences, Islamabad, Pakistan; United Nations Office for Project Services (UNOPS), UN Compound, Diplomatic Enclave, Islamabad, Pakistan

**Keywords:** Antenatal care, Barriers, Facilitating factors, Perceptions, Qualitative methods, Pakistan

## Abstract

**Background:**

World Health Organisation recommends that pregnant women with no complications should visit a healthcare provider at least four times to receive sufficient antenatal care services. In Pakistan only 37 % of women reported to have had four or more antenatal care visits during their last pregnancy. This study aimed to explore facilitators and barriers to use of antenatal care services in rural and urban communities of two selected districts in Pakistan.

**Methods:**

Qualitative explorative study using in-depth interviews with currently pregnant women, lady health workers and doctors providing antenatal care services, and focus group discussion with women who had a child aged 5 years or younger, was conducted in a rural community in the district Swabi and in a tertiary care hospital in urban Islamabad in Pakistan. The audio-recorded interviews and discussions were transcribed verbatim in Urdu (the language spoken by the respondents). A list of topical codes for all topics related to the research questions was developed. Subsequently the text pertaining to each topical code was discussed and summarised in a document that presented the findings for each topic using quotes and tables.

**Results:**

We conducted in-depth interviews with six lady health workers, four doctors, and ten currently pregnant women, and facilitated ten focus group discussions with women who had a child aged 5 years or younger. Currently pregnant women, and women who had a child aged 5 years or younger, were not aware of the recommended minimum number of antenatal care visits to be made during pregnancy. Facilitating factors to visit a particular health care facility were: availability of qualified healthcare providers (private facility); trust in healthcare providers; recommendation from a family member, friend or lady health worker (in rural areas); availability of good quality services including medical equipment and laboratory facilities; low cost (public facility); and easy access to the health facility (private facility). Common barriers to visiting a health facility for antenatal care services were: financial limitations; perceived absence of any major health problems during pregnancy; difficulties in reaching the health facility; restriction from husband or mother-in-law; busy performing household chores; no previous experience of antenatal care visits; and perceived unavailability of healthcare providers and/or services.

**Conclusions:**

The current study identified several policy-relevant facilitating factors and barriers to visiting a health facility for antenatal care services as reported by urban and rural women, and healthcare providers. There is a need to formulate and implement intervention packages based on these findings to increase the coverage of the recommended four antenatal care visits in Pakistan.

**Electronic supplementary material:**

The online version of this article (doi:10.1186/s12884-016-0829-8) contains supplementary material, which is available to authorized users.

## Background

The first 4 weeks of life – the neonatal period, is the most vulnerable time for a child’s survival. Globally in 2013, of 6.3 million under-five deaths, 2.8 million were neonatal deaths, accounting for 44 % of the under-five deaths [1]. The latest Pakistan Demographic and Health Survey (PDHS) 2012–13 reported a neonatal mortality rate of 55 per 1000 live-births, which is exceptionally high, and accounts for 62 % of under-five mortality [2]. To achieve national targets for Millennium Development Goal 4 (MDG-4) for child survival, it is important to substantially reduce neonatal deaths in Pakistan. Several socio-demographic factors have shown to be associated with neonatal mortality in Pakistan [3].

The Lancet 2005 series on neonatal survival highlighted that the use of antenatal care (ANC) services reduced all-cause neonatal mortality by 10–30 % while prematurity and low birth-weight by 20–55 % [4]. During ANC visits, women benefit from various interventions, including counselling about pregnancy complications, necessary laboratory investigations, the provision of iron/folic acid supplements, and tetanus toxoid vaccinations [5]. Several studies have shown a protective effect of ANC services on neonatal mortality [6–9]. World Health Organisation recommends that pregnant women with no complications should visit a healthcare provider at least four times to get sufficient ANC services [10]. Further, it is recommended to have the first ANC visit early, during the first trimester of pregnancy [10]. However, the use of ANC services is low in low- and middle-income countries due to several factors, such as, poor economic status, low educational levels, and lack of access to a facility [11–13].

In Pakistan, ANC services are provided by the maternal and child health services through the existing primary healthcare system including community health workers - the Lady Health Workers Programme, and static, public sector health facilities with subsidised medicines. The 2012–13 PDHS revealed that 76 % of women had at least one ANC visit during their last pregnancy 5 years prior to the survey. Further, 73 % of women received ANC services from skilled providers during their last pregnancy. However, only 37 % of women reported having four or more ANC visits while 42 % of women reported having their first visit during their first trimester of pregnancy. Furthermore, the percentage of women who had four or more ANC visits or who reported having their first ANC visit during the first trimester of pregnancy varied by place of residence, regions/ provinces, educational status and household wealth index [2]. Therefore, there is a need to implement interventions to improve ANC coverage by targeting marginal populations. Nevertheless, before the formulation of any interventions to improve the coverage ANC services, it is important to understand the community perceptions for the use of ANC services. This study aimed to explore facilitating factors and barriers to use of ANC services in rural and urban communities of two selected districts in Pakistan.

## Methods

### Study design

We conducted a qualitative explorative study to gather information from participants from the selected rural and urban areas in two districts of Pakistan. We employed face-to-face in-depth interviewing (IDI) and focus group discussion (FGD) methods to generate data. The detailed methodology has been presented in another publication, in which we reported the findings on the perceptions and practices relating to iron/folic acid supplementation during pregnancy [14].

### Ethical considerations

An informed written or verbal (based on the literacy status) consent was obtained from each of the participating individual in the current study. The human research ethics committees of the PIMS, Pakistan (‘Hospital Ethics Committee’) and the University of Sydney, Australia approved the study.

### Study sites, study population and selection of participants

The rural sample was selected from the district of Swabi while the urban part was conducted at the Pakistan Institute of Medical Sciences (PIMS), Islamabad. PIMS is the largest tertiary care, teaching hospital in Islamabad, the capital city of Pakistan. The institute has three major components – the Islamabad Hospital for adult patients with many sub specialties; the Children’s Hospital for paediatric patients; and the Maternal and Child Healthcare Centre for gynaecology and obstetrics patients.

The two study districts were selected based on predominant urban and rural settings, socioeconomic status and prevalence of use of ANC services. We collected information from: (a) urban and rural women who had a child aged 5 years or younger; (b) currently pregnant urban and rural women; (c) Lady Health Workers (LHWs) who were responsible for providing ANC services to rural pregnant women; and (d) doctors working at the PIMS Maternal and Child Health Centre, who provided ANC services to pregnant women visiting the PIMS Maternal and Child Health Centre.

In the rural settings, village leaders in each selected village were informed about the purpose of the study and their assistance sought for data collection. The village leaders and the Village Health Committee of the LHW Programme introduced two of the authors (YBN and BA) to the local LHW. The LHW assisted in locating currently pregnant women for IDIs and women who had a child aged 5 years or younger for FGDs.

In urban areas, we interviewed duty doctors who were providing ANC services at the outpatient department of the PIMS Maternal and Child Health Centre, Islamabad on the day of the interview. The currently pregnant women who visited the PIMS Maternal and Child Health Centre for ANC services were also interviewed. Women, who came with their sick children aged 5 years or younger to the PIMS Children’s Hospital, and who lived in Islamabad, were invited to participate in the FGDs. None of the invited participants from urban and rural setting refused to participate in the study.

### Sampling and selection of respondents

**Fig. 1:**
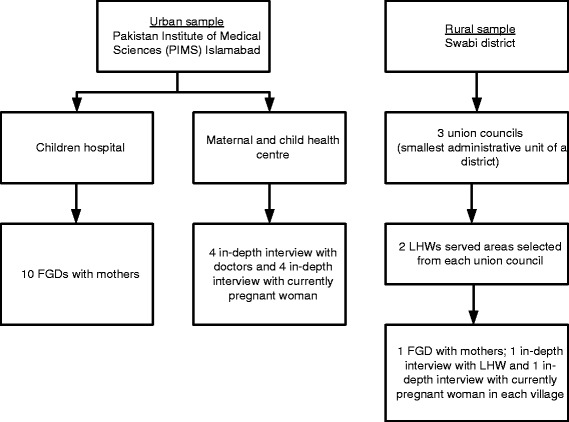
Sampling frame for the study

Sampling was purposive and the respondents were selected from the urban and the rural areas. Figure 1 summarises the sampling frame used in the study. We conducted six IDIs with LHWs, four IDIs with doctors, ten IDIs with currently pregnant women and ten FGDs with women who had a child aged 5 years or younger. In Swabi District, out of 56 union councils (the smallest administrative unit), we selected three union councils that were predominantly rural population, served by the LHW Programme, and had functioning first level government health facilities. From each union council we randomly selected two villages. In each selected village, IDIs with a local LHW and a currently pregnant woman were conducted. The currently pregnant woman was selected randomly from a list of currently pregnant women, which was prepared with assistance of the local LHW. A FGD with women who had a child aged 5 years or younger was conducted in each village. Six to eight women who were identified by the local LHW participated in each FGD.

In urban settings, we conducted IDIs with a duty doctor, and a currently pregnant woman who came for ANC on the day of the interview. We obtained a list of on-duty doctors on the day of the interview and a doctor was selected randomly for the IDI. Likewise, we obtained a list of women who came for ANC services and were still present at the waiting room on the day of interview from the registration counter of the PIMS Maternal and Child Health Centre and a pregnant woman was selected randomly for the IDI. To select women for FGDs in urban settings, we obtained a list of admitted children at the Isolation ward of the PIMS Children’s Hospital on the day of FGDs and 6 to 8 mothers who had children aged 5 years or younger were invited to participate in each FGD. The basic demographic profile of the participating currently pregnant women and women who had a child aged 5 years or younger is presented in Table 1. On the other hand, out of four selected doctors, one was working for the last 10 years, two were working for the last 4 years and one doctor was working for the last 1 year in the PIMS Maternal and Child Health Centre. Out of six selected LHWs, four were working for the last 15 years, two were working for 5 years and one LHW was working for the last 2 years in her community.

**Table 1:** Basic characteristics of respondents

Characteristics	Currently pregnant women^a^(*n* = 10)	Women who had child aged 5 years or younger^b^(*n* = 73)
	n (%)	n (%)
Age		
Less than 25	5 (50.0)	14 (19.2)
25 to 34 years	2 (20.0)	31 (42.5)
35 and more	3 (30.0)	28 (38.3)
Respondents educational status		
Illiterate	4 (40.0)	34 (46.6)
Primary	3 (30.0)	18 (24.6)
Secondary and above	3 (30.0)	21 (28.8)
Husband’s educational status		
Illiterate	3 (30.0)	25 (34.2)
Primary	2 (20.0)	23 (31.5)
Secondary and above	5 (50.0)	25 (34.2)
Duration of current pregnancy		
First trimester	2 (20.0)	NA
Second trimester	4 (40.0)	
Third trimester	4 (40.0)	
Number of children		
First time pregnant	3 (30.0)	NA
1 to 3	5 (50.0)	42 (57.5)
4 and more	2 (20.0)	31 (42.5)
Age of the youngest child		
First time pregnant	3 (30.0)	NA
Less than 18 months	4 (40.0)	27 (37.0)
18 to 36 months	3 (30.0)	27 (37.0)
More than 36 months	0 (0.0)	19 (26.0)

^a^Particpants of in-depth interviews. ^b^Participaants of focus group discussions. *NA* Not applicable.

### Data collection process

We developed separate guidelines for the FGD (Additional file 1: Guidelines for focus group discussion) with the women who had a child aged 5 years or younger and for the IDI (Additional file 2: Guidelines for indepth interview) with the currently pregnant women, LHWs and the doctors. The interview tools were translated into Urdu (the language spoken by the respondents). The data collection guidelines were pretested in the urban and the rural communities of the participating districts, but in an area where the actual study was not done, and were then modified on the basis of the pretest findings. The major topics contain perceptions about the use of ANC, its benefits/ disadvantages, and barriers and facilitating factors for ANC use. All interviews and discussions were conducted by two investigators (BA and YBN). FGDs were assisted by two social scientists who were hired and trained in the study objectives, methodology and the guidelines. As an example the following question was asked during interview: “In your opinion, what are the reasons to visit healthcare centres by pregnant women of your area?”

After obtaining informed consent, the session/interview was commenced according to the guidelines. A series of questions and probes were asked to understand the perceptions of participants about ANC services and their utilisation during pregnancy. We also explored the barriers to the use of ANC services in the participating communities. The average time for the FGD was 80 min, and 45 min for the IDI. An average of seven women who had a child aged 5 years or younger participated in FGDs. At the end of each session, information about the use of ANC services and its impact on maternal and newborn health was imparted to the participants. The data collection was conducted between August and November 2012.

### Data analysis

We audio recorded all interviews and discussions and took hand-written notes of the key information. We applied thematic approach that enabled the production of an ordered collective picture of rich data [15] for data analysis. Two investigators (YBN and BA), who collected the data, independently transcribed the recorded interviews and discussions verbatim and expanded the field notes in Urdu (the language spoken by the participants). Audio-recording and notes were then translated into English by the same investigators. A sample of them were discussed with other two investigators (AA and MJD) for quality check. We applied an inductive coding procedure where themes were derived empirically induced from the data that were related to our research questions [16]. YBN and BA developed a list of topical codes for all topics related to the research questions (barriers to and facilitators of ANC services utilisation) that was independently reviewed and verified by another investigator (AA) to ensure inter-coder reliability. Subsequently the text pertaining to each topical code was discussed among the researchers and summarised in a document that presented the findings for each topic using quotes and tables. NVivo version 10 (QSR International, Victoria Australia) was used to aid the data organisation and analyses. Data saturation was achieved during the data analysis.

## Results

### Perceptions and utilisation of ANC services

Almost all of the women perceived that the ANC services were important for the health of the mothers and the newborn babies. Further, they recommended pregnant women to receive ANC services. Urban women reported receiving ANC services for routine check-ups to prevent complications at the time of delivery, while a dominant perception of rural women was to obtain ANC services for the management of health-related problems during pregnancy. Almost all of the rural women were not aware of when to make first ANC visit and the timing of follow up visits. A substantial majority of the urban women were aware of having ANC visit as soon as possible after becoming pregnant. The urban women perceived that follow-up ANC visits were dependent on the health status of a pregnant woman and the advice of a healthcare provider. However, both the rural and the urban women were not aware of the recommended number of ANC visits in case of uncomplicated pregnancy.

*“During pregnancy, I think all women should go for check-up because we feel weakness. We get married in young age and after pregnancy we have many problems, for this reason it is important to go to health facility for treatment.”* (A rural mother: FGD participant).

The LHWs and doctors interviewed in this study mentioned that most of the pregnant women had a poor understanding and knowledge about ANC services. However, all of the LHWs stated that they visited all pregnant women in their catchment area every month and referred pregnant women to the nearest government health facility for ANC services or for management of any health-related problem during pregnancy. According to the LHWs, the common health-related problems requiring a referral were: severe vomiting, feeling nausea or dizziness, presence of pallor, any complications such as vaginal bleeding, swelling on legs, and inappropriate weight gain. However, the LHWs perceived that often due to lack of staff, medicines and difficulties to travel to a public health facility, some of the referred pregnant women went to a private clinic. Doctors participated in this study reported that many pregnant women visited the health facility for routine check-ups, usually in the second trimester of pregnancy. The doctors stated that they always advised pregnant women to revisit after a month. However, all of them perceived that some of the pregnant women visited the health facility once or twice for the management of any complications during their pregnancy.

*“Majority of them* (pregnant women) *visit us for their routine check-up as they are concern about their health. Some of them also visit us only in case of any problem and they never get back to us after that. Often these women come at the time of delivery due to a complication.”* (A doctor: IDI participant).

### Places, services and reasons for visiting a health facility for ANC

A few rural women but the majority of the urban women reported that during their last/ current pregnancy they went to a health facility for ANC services. However, the majority of the urban and the rural women who had ANC services in their last/ current pregnancy visited a private health facility. The reasons for visiting a private health facility for ANC services as reported by urban women included: availability of qualified and skilled providers, trust on healthcare providers at the private health facility, recommendation from a family member or a friend, availability of good quality services including medical equipments and pathology facilities, easy to reach a private health facility, and availability of an after-hours doctor. The reasons reported by urban women for visiting a public health facility for ANC services were: low cost as there was no consultation fee and often availability of free of cost medicines, the husband or the women herself was a government servant, and facilitation from a relative who was working at the public health facility. The availability of blood pressure monitoring equipment, pathology and ultrasonography services and prescriptions of medicines (including iron/folic acid supplements) were also important reasons for visiting a health facility for ANC services. The majority of the urban women reported that during their last visit to a health facility for ANC, they were examined by a doctor, weight and blood pressure were monitored (at private health facilities), they were advised about complications (at private health facilities), utilised pathology and radiology facilities, prescribed medicines/ supplements and were recommended for follow-up visits.

*“I went to a private clinic because it is convenient for us to visit a private clinic. It’s near my home and I went in the evening with my husband. Good quality laboratory facilities and medical equipments for monitoring of blood pressure and ultrasound are important for us during a health facility visit. It’s good to diagnose any condition which leads to complication later in pregnancy.”* (An urban pregnant woman: IDI participant).

On the other hand, the reasons for visiting a public health facility reported by rural pregnant women for ANC services were: low cost in terms of medicines and pathology services, being recommended by a local LHW, and trust in a government health system. On the other hand, the common reasons reported by the rural pregnant women for visiting a private health facility for ANC services were: easy to reach, recommendation from a family member, trust in healthcare provider and availability of ultrasonography and pathology services. The rural women stated that during their last visit to a health facility for ANC, the healthcare provider examined them, monitored their blood pressure (only at private health facilities), used pathology and radiology services (at private health facilities), medicines/ supplements were prescribed (at private health facilities) and were recommended for follow-up visits. However, few rural women stated that they were given information about complications or nutritional education.

*“We* (husband and myself) *go there* (health facility) *during pregnancy as it is near to our house and road leads to facility is in a good condition. She* (healthcare provider) *checks my blood pressure, urine and blood and provides medicines* (iron/folic supplements)*…I think she* (healthcare provider) *is a doctor and she has equipment to measure blood pressure. Many women go to her clinic.”* (A rural pregnant woman: IDI participant).

### Barriers to visiting a health facility for ANC services

Common reasons reported by the rural and the urban women for not visiting either a public or a private health facility for ANC services included: financial limitations including travelling cost and consultation fee in case of a private health facility, experienced or perceived absence of a major health problem during pregnancy, difficulties in reaching a government health facility in rural areas, restrictions from husband or mother-in-law to visit a health facility, busy in performing household chores, had no prior experience of ANC visit in earlier pregnancies, non-availability of staff and/or services at a public health facility and limited working hours of a public health facility. Some of the rural women did not go to a health facility because they were afraid of being diagnosed to have complications or complications related to hospitalisation. Few rural and urban women were also afraid of the side effects of medicines prescribed by a healthcare provider during ANC visit and, therefore, did not visit a health facility for ANC services.

*“I did not face any problems and no one ever told me to go to a health facility* (during pregnancy)*. It is also difficult for us to go to a private clinic as we are very poor people.”* (A rural mother: FGD participant).

The LHWs perceived that restrictions from the mothers-in-law, mothers or husbands as the most common reason for a pregnant woman not to visit a health facility for ANC services. Other reasons mentioned by the LHWs included not paying attention to the health of the women by their family and themselves, lack of transport to go to a public health facility and financial limitations to visit a private health facility. The doctors perceived lack of information about ANC services, business with household chores, restrictions from the mothers-in-law or other family members, lack of education and financial constraints to visit a private health facility as the major reasons for a pregnant woman not to visit a health facility.

*“Often poor women face restrictions especially from their mothers-in-law and do not come for check-up during pregnancy, unless they have any major complications. Lack of education is also a major reason.”* (A doctor: IDI participant).

### Intentions to use of ANC services in the future

The majority of both the rural and the urban women expressed their intension to visit a public health facility for ANC services during their future pregnancies, if a skilled healthcare provider, essential medical equipment and free of cost medicines were available at the facility. Further, a few rural women suggested that government and other non-government organisations working in their areas should provide them funds for transportation to visit a government health facility for ANC services. In rural areas, the LHWs were hopeful that with their efforts to increase awareness in the community about the importance of ANC, availability of services at public health facilities by the government, and the support from family members, a substantial majority of the pregnant women would visit a government health facility for ANC service. However, in urban areas, the doctors were not sure about how to improve the coverage of ANC and suggested that the government along with other international donor agencies should conduct awareness campaigns through mass media about the importance of ANC, increase the number of skilled health providers, and provide all essential medicines and pathology services free of cost at the government health facilities to improve ANC coverage.

*“I am working in my village for the last 15 years and there is an improvement in health behaviour of people of my village. I am hopeful that once they will be aware of benefits of visiting a health facility during pregnancy, which I am trying to impart, more and more pregnant women will visit health facilities during pregnancy.”* (A LHW: IDI participant).

## Discussion

### Main findings and their significance

We found that the most common reasons for a pregnant woman to visit a health facility for ANC services were: the presence of qualified and skilled healthcare providers (private facility), trust in the healthcare providers, recommended by a family member, friend or a local LHW, low cost due to free of cost medicines (public facility), availability of good quality services (private facility), facilitation by a relatives at the health facility (public facility), easy to reach the health facility (private facility), and availability of healthcare providers even in evening hours (private facility). The barriers for a pregnant woman to visit a health facility for ANC services were: financial limitations, experienced or perceived absence of a major health problem during pregnancy, difficulties in reaching a government health facility in rural areas, restrictions from husband or mother-in-law to visit a health facility, perceived lack of information about ANC services, busy in performing household chores, had no prior experience of ANC visit in previous pregnancies, experienced or perceived non-availability of staff and/or services at the health centre (public facility) and limited working hours of the health facility (public facility). The current study findings which highlighted the facilitating factors and barriers to use ANC services are important for policymakers and programme managers working in the field of maternal, newborn and child health in Pakistan. These findings are also important for researchers to formulate intervention packages to improve the use of ANC services coverage in low- and middle-income countries like Pakistan.

### Comparison with other studies

The findings of this study are consistent with other studies carried out to investigate the reasons for low coverage of use of ANC services. An ethnographic study from Pakistan reported lack of financial support, restriction from family members and lack of transportation to reach a health facility for ANC services were the major reasons for non-use of ANC services [17]. Another study from the least developed province of Pakistan – Balochistan, found that distance from a health facility with lack of transportation, low family income, unavailability of healthcare providers, medicines and other essential services at a public health facility, and not having adequate awareness about ANC services were the main reasons for a pregnant woman not to visit a health facility for ANC services [18]. A study from Zimbabwe found that pregnant women did not visit a health facility for ANC services due to long distance and scarce transportation, financial limitations, difficulties in crossing big rivers during the rainy season, shame to visit a health facility with torn clothes or tight dresses, shame for having too many pregnancies or becoming pregnant at older age, negative attitudes of healthcare providers, long waiting time and poor quality of care at the facility [19]. A recent study from Afghanistan found that the low motivation, lack of support from husbands or mothers-in-law, poor economic conditions of the family, difficulties with transportation to reach a health facility and being treated poorly by healthcare providers were the common reasons for lack of use of ANC services [20]. A study from Indonesia reported the main reasons for attending ANC services were recommendation by the family members, to get assurance of safe health of mothers and newborns, and to receive treatment for health-related problems during pregnancy. Moreover, the authors reported that the family’s economic problems, difficulty in reaching a health facility, presence of no major health-related problems during pregnancy, and limited availability of health services were the major reasons for non-use of ANC services [21].

### Programmatic implications

The provision of ANC services to all pregnant women is one of the essential components of Pakistan’s National Health Policy [22]. In rural areas, the LHWs provide the primary healthcare services to pregnant women through their home visits. In addition, they also routinely refer pregnant women to nearby first level government health facilities to receive ANC services from a skilled healthcare provider [23]. However, visits to these facilities for ANC services is low due to many reasons, such as restricted hours of operation, often located far from the target population, unavailability of a skilled healthcare provider [24] and often due to lack of awareness of the community about the availability of ANC services [18]. There is a need to formulate and implement a package of interventions, such as community awareness campaigns, influence the behaviour of husbands and/or mothers-in-law to encourage pregnant women to access ANC services, provision of travelling cost or improvement in transportation facilities, and availability of skilled healthcare providers, free of cost or subsidised medicines and adjustment of working hours of public health facilities. The proposed interventions can be tested in community based trials in various regions across the country through public-private partnership. Communication strategies such as women’s group meetings and mobile phone services have shown a positive effect on ANC coverage in Nepal [25] and Tanzania [26]. In Pakistan, the LHWs are conducting women’s group meetings in their catchment areas each month to raise awareness around key maternal and child health issues. However, to improve the effectiveness of these group meetings, there is a need to enhance the communication skills of the LHWs, and provide them supportive supervision.

### Strengths and limitations

The major strength of the current study was that we collected information in two districts of Pakistan with urban and rural settings from diverse categories of respondents, that is, women who had a child aged 5 year or younger, currently pregnant women, the LHWs in rural communities and the doctors in urban areas providing ANC services. However, as a qualitative research, the results were not intended to be representative of all districts in Pakistan. Further, using various data collection techniques in our study – FGDs and IDIs, provided us a better opportunity to clearly understand the problems from different perspectives covering those of the recipients and providers, both in rural and urban settings. However, inclusion of the family members of the women in the study would have provided a more comprehensive understanding of the barriers to ANC coverage and that could be achieved in a future study. Further, we did not determine the quality of counselling and information imparted by the healthcare providers by a direct observation at the time of ANC visit. Hence, further investigation is needed to examine these issues.

## Conclusions

This study highlighted several facilitating factors and barriers to visiting a health facility for ANC services by pregnant women in rural and urban communities of two selected districts in Pakistan. The findings indicate a need of formulation and testing of interventions, especially in rural communities, to improve ANC services coverage. This will, subsequently, help in reducing neonatal mortality and achieving the national targets of MDG-4 for child survival in Pakistan and other similar low-and middle-income countries.

## Additional files

Additional file 1:
**Guideline for focus group discussion.** (DOCX 27 kb) Additional file 2:
**Guideline for** **in-depth** **interview.** (DOCX 26 kb)

## Abbreviations

ANC: Antenatal care
FGD: Focus group discussion
IDI: In-depth interview
LHW: Lady health worker
MDG: Millennium development goal
PDHS: Pakistan Demographic and Health Survey
PIMS: Pakistan Institute of Medical Sciences
