# Supplementary material for: Qualitative exploration of facilitating factors and barriers to use of antenatal care services by pregnant women in urban and rural settings in Pakistan
Source: BMC Pregnancy Childbirth. 2016 Mar 1;16:42. doi: 10.1186/s12884-016-0829-8 (PMC4772650; doi:10.1186/s12884-016-0829-8)
Supplement: Additional file 1: — Guideline for focus group discussion. (DOCX 27 kb) [file 12884_2016_829_MOESM1_ESM.docx]

Guideline for focus group discussion

| General instructions for the facilitator | | | | | | | | |  |
| --- | --- | --- | --- | --- | --- | --- | --- | --- | --- |
| - First, introduce yourself and the note taker and tell the respondents the purpose of the study, approximate duration of the group discussion and outcome of the discussion and read out the consent form. - Afterwards, take the verbal consent and handover the participant information sheet. - Inform every participants about the digital audio recording of the discussion and obtain their consent for audio recording too. - If all of them refuse to get their discussion be recorded or equipment is not working properly, please continue with the discussion without recording and take elaborate notes. - If they agree with audio recording of the discussion, kindly check the equipment before the start of the discussion. - Ask the respondents if they want to ask any question before the start of the discussion. | | | | | | | | |  |
| Questions | | | | | | | | |  |
| - Questions given in this tool will be used for focus group discussion with mothers. - Kindly go through each section one by one. First introduce the topic of the section and then generate discussion. Instructions for each section and/or for questions are printed as bold and italic. - Kindly elaborate and explain each question before getting answer. - Kindly write down the key points of the answer and keep on recording the discussion. - At the end of the discussion, thank the participants. | | | | | | | | |  |
| TO BE FILLED BY THE FACILITATOR | | | | | | | | |  |
| Name of facilitator | | |  | | Name of note taker |  | | |  |
| Date of interview | | | __ __/__ __/__ __ | | Time of start of interview | __ __\|__ __ ***(in 24 hours format)*** | | |  |
| Time of end of interview | | | __ __\|__ __ | | Audio recording done? | YES | | NO |  |
| ***If NO,*** Why? | | |  | | | Address |  | |  |
| **Woman no.** | **Age** | | **No. of children** | **Age of youngest child (in months)** | | | **Highest qualification** | | |
| 1 |  |  |  |  | | |  | | |
| 2 |  |  |  |  | | |  | | |
| 3 |  |  |  |  | | |  | | |
| 4 |  |  |  |  | | |  | | |
| 5 |  |  |  |  | | |  | | |
| 6 |  |  |  |  | | |  | | |
| 7 |  |  |  |  | | |  | | |
| 8 |  |  |  |  | | |  | | |
| 9 |  |  |  |  | | |  | | |
| 10 |  |  |  |  | | |  | | |
| 11 |  |  |  |  | | |  | | |
| 12 |  |  |  |  | | |  | | |

| **SECTION B: KNOWLEDGE AND PRACTICE ABOUT ANTENATAL (DURING PREGNANCY) CARE AND CHILD BIRTH** | |
| --- | --- |
| ***The purpose of this section is to investigate about the utilization of antenatal care (during pregnancy) and child birth services by pregnant women during her current pregnancy.*** | |
| B1 | What do you think, during pregnancy, do pregnant women feel any changes in terms of their health. What are these changes and why?  ***Encourage them to share their experience of their most recent pregnancy.*** |
| B2 | In your opinion, what dietary habits a pregnant woman follows during her pregnancy?   - Why - What are food taboos? - Advantages/disadvantages of these habits? |
| B3 | Do any of you follow any rules regarding food and mobility during your most recent pregnancy?   - Why? - Who enforce you? - Are these rules similar in the entire area or may differ across socio-economic & cultural groups? |
| B4 | In your opinion, what various healthcare services a pregnant woman need?   - What are the different health services a pregnant woman needs during her pregnancy? (probe components of the services) - Did any of you receive these health services during your pregnancy? ***ask them to share their experience.*** - Where and how many times did you receive them? - How much did they cost? - What are the advantages and disadvantages of these health services (for the mother, child and family)? |
| B5 | About what proportion of pregnant women in your area/ neighbourhood go to the health facility to take services during pregnancy.   - Why some women do not visit facilities for services during pregnancy? - What are the possible reasons ***[Probe: workload, poverty, cultural restriction on going out during pregnancy, mother-in-law/husband does not want etc]?*** |
| B6 | Who generally assisted mothers during childbirth in your neighbourhood/ family members? Please provide me list of persons/ institutes who provide assistance during child birth.   - Why? |
| **SECTION C: KNOWLEDGE AND PRACTICE ABOUT IRON-FOLIC ACID SUPPLEMENTS** | |
| ***The purpose of this section is to access the current knowledge and practice of mothers about iron-folic acid supplements used during pregnancy.*** | |
| C1 | Have you ever heard about iron-folic acid supplements? Do you have a local name for iron-folic acid supplement? Please describe the names. |
| C2 | What do you know about use of iron-folic acid supplements during pregnancy?  ***Ask her advantages and/or disadvantages of use of iron folic acid during pregnancy.*** |
| C3 | Have you ever received information about iron-folic acid supplement?   - Why? - From who? - What was the information you received? |
| C4 | We know there might be some people who may not use medicines they are advised by the doctors/health workers or do not follow the instruction about taking the medicines? Are there women in your area/ neighbourhood who do not use or refused to use iron-folic supplements? Please tell me who are they (NO need of names)?   - Are they from any specific socio-economic groups? - Why do you think they are not using/ have refused to use iron-folic acid supplementation? Please describe***. [Interviewers: probe for the following if the respondents does not talk about them spontaneously]:*** - Lack of about iron-folic acid requirement - Lack of knowledge about iron-folic acid supplementation program - They don’t like it - Because of the previous experience of side-effects - Fear of side effects - Other family members don’t let them to use; why? - Money involved |
| C5 | Have you any of you ever received iron supplement?   - Why? - Where? - Who gave? - Any payment? How much? - How many tablets? - When? current pregnancy or other pregnancies (1st, 2nd, 3rd trimester?) - Any advice or information received? what? |
| C6 | Have any of you ever taken iron/folic acid supplements during pregnancy?   - Why? - When? (2nd or 3rd trimester)? - Time to take? Any or Morning, afternoon, evening? - How? (e.g. mix it with orange juice or take it together with tea, coffee?) - Do you feel some side effects? what? how do you deal? - How long have been taking this tablet? - Do you usually take iron/folic acid tablet daily? why? - What are the advantages/disadvantages of use of iron during pregnancy? |
| C7 | Do you think iron-folic acid has a role in the health of pregnant woman, the foetus and health of the newborn babies. What role does it play? |
| **SECTION D: KNOWLEDGE ABOUT MATERNAL ANAEMIA** | |
| ***The purpose of this section is to know the current knowledge of a pregnant woman maternal anaemia, its sign and symptom, causes and treatment.*** | |
| D1 | Have any of you ever heard of Anaemia? Do any of you have local names for Anaemia? Please describe the names. |
| D2 | Have any of you ever received information about Anaemia?  ***If 'YES' then ask,***   - From who? - From where did you get information about Anaemia? - What is the information given? |
| D3 | ***If anyone is aware of anaemia then ask:***   - Please tell me what you know about anaemia. - What are the Signs and symptoms of anaemia? - Causes and consequences of Anaemia? - Treatment? |
| D4 | Did any of you ever diagnosed by the health worker/ doctor to be anaemic during your pregnancy? ***[explain it by elaborating the common sign and symptoms of anaemia]***   - Who told you? - What sign and symptoms you experienced? - How did the health personnel/any other person know that you are anaemic? Did they perform some tests? - What advice given to you? - Are you taking any medicines? - Do you feel better after taking medicine? |
| D5 | Do you think a woman with this problem should go to health facility? Why |
