# Supplementary material for: Qualitative exploration of facilitating factors and barriers to use of antenatal care services by pregnant women in urban and rural settings in Pakistan
Source: BMC Pregnancy Childbirth. 2016 Mar 1;16:42. doi: 10.1186/s12884-016-0829-8 (PMC4772650; doi:10.1186/s12884-016-0829-8)
Supplement: Additional file 2: — Guideline for in-depth interview. (DOCX 26 kb) [file 12884_2016_829_MOESM2_ESM.docx]

Guideline for in-depth interview

| General instructions for the Interviewer | | | | | | | | | | | | | | | | |
| --- | --- | --- | --- | --- | --- | --- | --- | --- | --- | --- | --- | --- | --- | --- | --- | --- |
| - First, introduce yourself and tell the respondent the purpose of the study, approximate duration of the interview and outcome of the interview and read out the consent form. - Afterwards, take the verbal consent and handover the participant information sheet. - Inform her about the digital audio recording of the interview and obtain her consent for audio recording too. - If she refuses to get her interview be recorded or equipment is not working properly, please continue with the interview without recording and take elaborate notes. - If she agrees with audio recording of the interview, kindly check the equipment before the start of the interview. - Ask the respondent if she wants to ask any question before the start of the interview. | | | | | | | | | | | | | | | | |
| Questions | | | | | | | | | | | | | | | | |
| - Questions given in this tool will be used for in-depth interview with currently pregnant woman. - Kindly go through each section one by one. First introduce the topic of the section and then ask each question. Instructions for each section and/or for questions are printed as bold and italic. - Kindly elaborate and explain each question before getting answer. - Kindly write down the key points of the answer and keep on recording the interview. - At the end of the interview, thank the interviewee and fill out the observation sheet. | | | | | | | | | | | | | | | | |
| TO BE FILLED BY THE INTERVIEWER | | | | | | | | | | | | | | | | |
| Name of interviewer | | |  | | | | | | | | Date of interview | | | __ __/__ __/__ __ | | |
| Time of start of interview | | | __ __\|__ __ ***(in 24 hours format)*** | | | | | | | | Time of end of interview | | | __ __\|__ __ | | |
| Is audio recording done? | | | YES | NO | ***If NO,*** Why? | | |  | | | | | | | | |
| Is interview completed? | | | YES | NO | ***If NO,*** Why? | | |  | | | | | | | | |
| **SECTION A: SOCIO-DEMOGRAPHIC CHARACTERISTICS OF PREGNANT WOMAN** | | | | | | | | | | | | | | | | |
| A1 | What is your name? | | |  | | | | | | | | A2 | What is your age? | |  |  |
| A3 | What is your occupation? | | |  | | | A4 | | What is your highest level of education? | | | |  | | | |
| A5 | What is your husband's highest level of education? | | |  | | | A6 | | What is your husband's occupation? | | | |  | | | |
| A7 | No. of pregnancies before this pregnancy | | | | |  |  | | A8 | Duration of your pregnancy in months | | | | |  |  |
| A9 | Address |  | | | | | | | | | | | | | | |

| **SECTION B: KNOWLEDGE AND PRACTICE ABOUT ANTENATAL (DURING PREGNANCY) CARE AND CHILD BIRTH** | |
| --- | --- |
| ***The purpose of this section is to investigate about the utilization of antenatal care (during pregnancy) and child birth services by pregnant women during her current pregnancy.*** | |
| B1 | During your current pregnancy, are you feeling any changes in terms of your:   - Health? what are these? - Why?   ***Ask her experience with current pregnancy in terms of health, also ask her why.*** |
| B2 | In your opinion, what dietary habits a pregnant woman follows during her pregnancy?   - Why - What are food taboos? - Advantages/disadvantages of these habits? |
| B3 | Do you follow any rules regarding food and mobility during pregnancy?   - Why? - Who enforce you? - Are these rules similar in the entire area or may differ across socio-economic & cultural groups? |
| B4 | In your opinion, what various healthcare services a pregnant woman need? ***make a list of all services.***   - What are the different health services a pregnant woman needs during her pregnancy? (probe components of the services) - What services are needed by you? - Did you receive these health services during your pregnancy? - Where and how many times did you receive them? - How much did they cost? - What are the advantages and disadvantages of these health services (for the mother, child and family)? |
| B5 | About what proportion of pregnant women in your area/ neighbourhood go to the health facility to take services during pregnancy.   - Why some women do not visit facilities for services during pregnancy? - What are the possible reasons ***[Probe: workload, poverty, cultural restriction on going out during pregnancy, mother-in-law/husband does not want etc]?*** |
| B6 | Who generally assisted mothers during childbirth in your neighbourhood/ family members? Please provide me list of persons/ institutes who provide assistance during child birth.   - Why? - Where will you go for delivery? Why? |
| **SECTION C: KNOWLEDGE AND PRACTICE ABOUT IRON-FOLIC ACID SUPPLEMENTS** | |
| ***The purpose of this section is to access the current knowledge and practice of pregnant women about iron-folic acid supplements used during pregnancy.*** | |
| C1 | Have you ever heard about iron-folic acid supplements? Do you have a local name for iron-folic acid supplement? Please describe the names. |
| C2 | What do you know about use of iron-folic acid supplements during pregnancy?  ***Ask her advantages and/or disadvantages of use of iron folic acid during pregnancy.*** |
| C3 | Have you ever received information about iron-folic acid tablet?   - Why? - From who? - What was the information you received? |
| C4 | Are there women in your area/ village who do not use or refused to use iron-folic supplements? Please tell me who are they (NO need of names)?   - Are they from any specific socio-economic groups? - Why do you think they are not using/ have refused to use iron-folic acid supplementation? Please describe***. [Interviewers: probe for the following if the respondents does not talk about them spontaneously]:*** - Lack of about iron-folic acid requirement - Lack of knowledge about iron-folic acid supplementation program - They don’t like it - Because of the previous experience of side-effects - Fear of side effects - Other family members don’t let them to use; why? - Money involved |
| C5 | Have you ever received iron supplement?   - Why? - Where? - Who gave? - Any payment? How much? - How many tablets? - When? current pregnancy or other pregnancies (1st, 2nd, 3rd trimester?) - Any advice or information received? what? |
| C6 | Some pregnant women use iron supplements during their pregnancy. Are you taking iron/folic acid tablet during your current pregnancy?   - Why? - When? (2nd or 3rd trimester)? - Time to take? Any or Morning, afternoon, evening? - How? (e.g. mix it with orange juice or take it together with tea, coffee?) - Do you feel some side effects? what? how do you deal? - How long have been taking this tablet? - Do you usually take iron/folic acid tablet daily? why? - What are the advantages/disadvantages of use of iron during pregnancy? |
| C7 | Do you think iron-folic acid has a role in the health of pregnant woman, the foetus and health of the newborn babies. What role does it play? |
| **SECTION D: KNOWLEDGE ABOUT MATERNAL ANAEMIA** | |
| ***The purpose of this section is to know the current knowledge of a pregnant woman maternal anaemia, its sign and symptom, causes and treatment.*** | |
| D1 | Have you ever heard of Anaemia? Do you have local names for Anaemia? Please describe the names. |
| D2 | Have you ever received information about Anaemia?  ***If 'YES' then ask,***   - From who? - From where did you get information about Anaemia? - What is the information given? |
| D3 | ***If she is aware of anaemia then ask her:***   - Please tell me what you know about anaemia. - What are the Signs and symptoms of anaemia? - Causes and consequences of Anaemia? - Treatment? |
| D4 | Did anyone told you that you are anaemic during your pregnancy? ***[explain it by elaborating the common sign and symptoms of anaemia]***   - Who told you? - What sign and symptoms you experienced? - How did the health personnel/any other person know that you are anaemic? Did they perform some tests? - What advice given to you? - Are you taking any medicines? - Do you feel better after taking medicine? |
| D5 | Do you think a woman with this problem should go to health facility? Why |
